# Next-generation viral detection through AI-enhanced nanotechnology: advances, challenges, and future directions

**DOI:** 10.3389/fmolb.2026.1750124

**Published:** 2026-03-18

**Authors:** Pankaj Garg, Gargi Singhal, Sharad S. Singhal

**Affiliations:** 1 Department of Chemistry, GLA University, Mathura, Uttar Pradesh, India; 2 Undergraduate Medical Sciences, S.N. Medical College Agra, Agra, Uttar Pradesh, India; 3 Department of Medical Oncology and Therapeutics Research, Beckman Research Institute of City of Hope, Comprehensive Cancer Center and National Medical Center, Duarte, CA, United States

**Keywords:** artificial intelligence, deep-learning, nanotechnology, virus prediction, biosensors, smart health systems, real-time monitoring

## Abstract

Emerging viral outbreaks, such as the COVID-19 pandemic, have highlighted the critical need for rapid, accurate, and scalable virus detection systems. This review aims to explore the integration of artificial intelligence (AI) and nanotechnology as a transformative approach for real-time virus prediction, monitoring, and management. The review systematically analyzes how machine learning (ML) and deep learning (DL) algorithms are being applied to identify viral mutations, forecast outbreak trajectories, and analyze complex virological data. It also highlights recent advances in nanotechnology, including the development of nanosensors, nanoparticle-based diagnostics, and lab-on-chip devices. The synergy between AI and nanotechnology is examined through selected case studies and near-real-world implementation efforts. The convergence of AI and nanotechnology represents a promising translational pipeline toward highly sensitive, rapid, and personalized viral detection systems, with substantial clinical validation and regulatory maturation still required before routine deployment. When combined, AI enhances the interpretability and responsiveness of nanotech-based diagnostics, while nanodevices provide high-resolution data for AI-driven prediction models. This integration supports more adaptive, data-driven public health responses. This review presents an up-to-date, interdisciplinary overview of AI–nanotech integration in virology. It identifies current challenges such as data privacy, algorithmic bias, and regulatory barriers, while proposing future directions for personalized and globally inclusive virus surveillance systems. The combined power of biological insight and technological innovation outlines an emerging paradigm for managing viral threats, contingent upon continued translational validation and real-world implementation.

## Graphical Abstract

**Figure:**
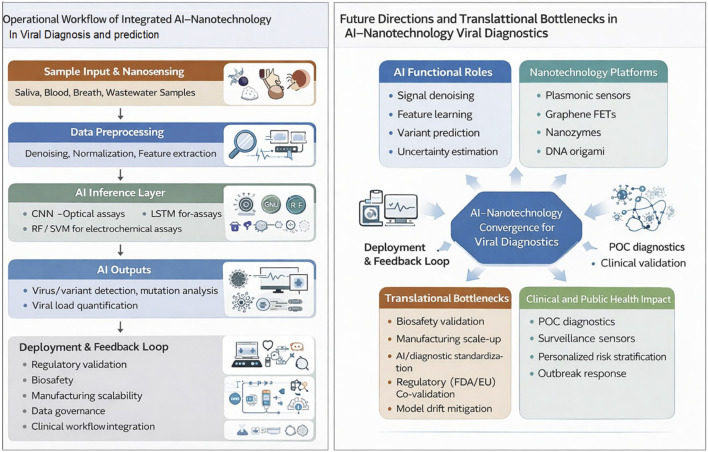

## 1 Introduction

**FIGURE 1:**
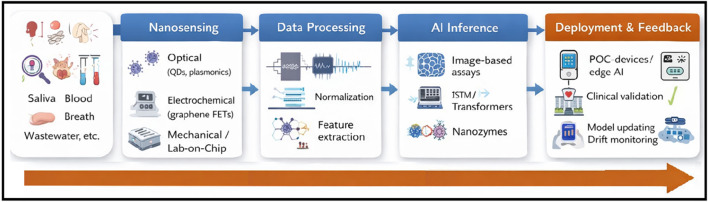
Operational workflow of AI–nanotechnology–integrated viral diagnostics. Schematic representation of the end-to-end diagnostic pipeline spanning biological sampling (e.g., saliva, blood, breath condensate, wastewater), nanosensing modalities (optical plasmonic or QD–based sensors, electrochemical graphene field-effect transistor biosensors, and lab-on-chip microfluidic platforms), data preprocessing (noise filtering, normalization, and feature extraction), AI-based inference (CNNs for image-based readouts and LSTM/Transformer architectures for biosensor time-series data), and deployment with clinical feedback (point-of-care or edge-AI devices, clinical validation, and post-deployment model updating with drift monitoring). The workflow highlights how nanosensor-derived biosignatures are transformed into actionable diagnostic outputs within a translational pipeline.

The emergence of the COVID-19 pandemic has encouraged researchers to come up with helpful technologies that can detect, trace, and predict certain viruses quickly. Conventional viral diagnostics remain effective but are often resource-intensive and slow to adapt to rapidly evolving pathogens. To overcome these problems, researchers are starting to use the connection between artificial intelligence (AI) and nanotechnology (Gawande et al., 2025). Due to its huge data analysis skills, AI is transforming virology by helping predict mutations, plan for outbreaks, and study host and pathogen relations (Padhi et al., 2023). In addition, nanotechnology helps to identify biological agents by spotting nucleic acids on the molecular scale with nanosensors, quantum dots, and lab-on-chip systems (Zhang et al., 2024). The current study gives an overview of using AI and nanotechnology together to predict and identify viruses, discussing main concepts, current best applications, real case studies, and future challenges. The integrated workflow of this AI–nanotechnology approach is illustrated in (Figure 1). This Perspective focuses on the translational pathway from nanosensor signal acquisition to AI-driven clinical interpretation across laboratory, point-of-care, and surveillance settings (Adir et al., 2020).

Since viral outbreaks are emerging more frequently, the first step in controlling diseases is to detect pathogens early on. If a disease is identified early, it is possible to act fast with medical care and containment solutions to reduce both the transmission and seriousness of the disease. Centralized laboratory-based diagnostics can delay actionable responses during the early phase of outbreaks (Hu et al., 2024). However, instantly, on-site diagnostics using AI and nanotechnology can reduce response times by a considerable margin. Because of these technologies, health authorities can act earlier in a pandemic by enabling decentralized screening and rapid triage (Chang et al., 2021).

AI enables learning from viral genomes and epidemiological trends, while machine learning (ML) identifies patterns in viral evolution, transmission, and host interaction relevant to public health decision-making. Additionally, nanotechnology enables rapid detection of viral proteins and nucleic acids using nanosensors and lab-on-chip systems (Bowyer et al., 2025). AI-assisted interpretation of nanosensor outputs improves sensitivity, specificity, and robustness of point-of-care diagnostics (Das and Chandra, 2023).

AI–nanotechnology convergence supports adaptive surveillance as viral variants and transmission patterns progressed. Data collected from nanoscale sensors can be analyzed by AI to track abnormalities, forecast outbreak trajectories, and inform prevention strategies (Singh et al., 2017). Wearable nanosensors integrated with AI may enable continuous personal-level monitoring and early warning (Suvvari and Kandi, 2024). Unlike prior descriptive reviews, this Perspective proposes a concrete operational framework for AI–nanotechnology convergence, outlining how nanosensor data streams are computationally transformed into clinically actionable predictions across laboratory, point-of-care, and public health surveillance settings. While the COVID-19 pandemic provides an illustrative real-world anchor due to unprecedented data availability and translational acceleration, the proposed framework is virus-agnostic and applicable to diverse viral systems including influenza, HIV, HPV, dengue, and RSV.

## 2 Overview of AI techniques in virology

In virology, AI applications rely on supervised learning to make models that are structured and easy to understand using available labeled data. Depending on the study, researchers often classify viral strains, determine disease outcomes, and spot significant mutations related to the disease by using Support Vector Machines (SVMs), decision trees and Random Forests (RF). Annotated datasets allow researchers to use these models to help separate infected from non-infected individuals and people with mild and severe symptoms of disease (Elste et al., 2024). Because of supervised models, researchers can better choose the genetics and clinical factors that are most influential in shaping viral behavior. DL systems, specifically convolutional neural networks (CNNs), recurrent neural networks (RNNs), long short-term memory networks (LSTMs) and transformers, allow researchers to effectively deal with complicated viral data. CNNs are skilled at finding structural patterns in viruses, while LSTMs and transformers work well when it comes to understanding the time-related changes in viruses. They can be set to detect changes in genomic sequences linked to pathogens and medications and are used in related work such as processing genetic information, diagnosing viruses from images, and predicting the transmission of epidemics (Yue et al., 2023).

Natural language processing (NLP) tools have made the annotation of genomes and mining of literature much more automated. NLP technology helps to explore genetics by processing unstructured text about genes to recognize their function, identify open reading frames, and sort viral variants using their traits. NLP tools help speed up compiling information from new reports, scientific journals, gene databases, and clinical studies in virology research (Liu et al., 2011). Reinforcement learning is another approach in this area, which is currently being explored for predicting the progression of viral infections and testing different interventions. In these models, the AI is trained by investigating different actions (for example, setting vaccination dates or enforcing restrictions on movement) in a simulated version of how a disease spreads. This way of working is very helpful for making flexible plans that can adapt as trends in the outbreak change, providing insights into how to effectively slow down the spread of a virus (Weltz et al., 2022). Studying virus-host interactions with the help of AI is a major breakthrough. This depends on combining various datasets such as transcriptomics, proteomics, and genomics to identify how viruses take control of the host cell organisms.

Analyzing the interactions shared by viruses and hosts, AI can suggest new therapies and the basis for predicting zoonotic spillover. Experts use graph neural networks and matrix factorization models to analyze data and understand the relationships inside complex biological systems. The choice of AI model in nanosensor-based viral diagnostics is not influenced by the novelty of the algorithm but based on the signal modality of a biosensor. CNNs are specifically designed to enable image-based nanoparticle and fluorescence readout whereas the long short-term memory networks (LSTMs) and transformer architectures are best adapted to continuous biosensor time-series streams produced by wearable or environmental nanosensors. In low-data regimes, ensemble models are powerful and interpretable nanosensors due to the electrochemical nanosensors they are useful with, like random forests and support vector machines (SVMs). Incorporation of deep learning (DL) of feature-engineered ML models with legacy pipelines may achieve an additional enhancement of generalization in point-of-care settings with noisy and real-world conditions (Pan et al., 2012). The diverse AI models applied in virology are summarized in Table 1, highlighting their application areas, tools, and key advantages.

**TABLE 1:** Overview on the role of AI techniques in virus detection.

AI technique	Application area	Examples/Tools	Key benefits	References
Supervised Learning	Classification of viral genomes, subtype identification	SVM, Random Forest	Enables precise categorization based on labeled data	Elste et al. (2024), Gao and Liu (2024)
Deep Learning (CNN, RNN, LSTM, Transformers)	Viral image recognition, genome pattern prediction	ViraMiner, DeepVirus	Learns complex data features automatically for high accuracy	Yue et al. (2023), Azevedo et al. (2024)
NLP (Natural Language Processing)	Genome annotation, biomedical literature extraction	BioBERT, SciSpacy	Transforms unstructured text into meaningful insights	Liu et al. (2011), Al-Garadi et al. (2022)
Reinforcement Learning	Modeling viral spread, simulating public health interventions	Custom simulation frameworks	Adapts to changing environments to optimize outcomes	Weltz et al. (2022), Bushaj et al. (2022)
Virus–Host Interaction Prediction	Mapping protein–protein interactions	DeepViral, AlphaFold	Aids in uncovering disease mechanisms and targets	Pan et al. (2012)

## 3 Viral genome analysis and mutation prediction

AI analysis assists in studying viral genomes and predicting possible changes in how spreadable and treatable the pathogen is. By studying sequencing information, ML algorithms can quickly find and categorize viral variants. With these tools, mutations related to virulence or evading the immune system can be detected, making it possible to quickly modify current diagnostics, treatments, and vaccines (Gao and Liu, 2024). AI tools in genomic analysis played a big part in spotting and tracking SARS-CoV-2 variants Delta and Omicron, while helping public health officials react globally. Generative models, for example Generative Adversarial Networks (GANs) and Variational Autoencoders (VAEs), are pioneering ways to predict which areas of DNA will be most likely to mutate. They can find patterns in the virus’s genome and forecast possible paths of evolution. Generative models allow scientists to study and predict the possible changes that a virus could go through by using synthetic data, which helps them to develop solutions before the virus mutates. Host receptor binding is reported to be influenced by structural changes in viral proteins, and GANs have been instrumental in modeling such alterations (Bagabir et al., 2022).

Seasonal flu and possible pandemics are made more likely by antigenic drift and shift, and AI helps researchers predict these important developments. In predictive models, changes in viral antigenic sites over time are simulated by researchers, which gives vaccine makers an edge in forecasting possible future strains. This approach lowers the chance of vaccine/strain mismatches and improves the results of immunization (Perofsky et al., 2024). Many advanced systems and tools are now available to help analyze AI-driven viral genomes. DeepVirus can spot viruses from the input sequence data by using DL algorithms, whereas ViraMiner deploys CNNs to detect viral sequences within human samples with extra care for accuracy. These models illustrate how AI converts genomic data into information that helps us watch for, predict, and handle viral threats right away (Azevedo et al., 2024).

Outside of coronaviruses, DL–based genomic surveillance has also been widely used to predict antigenic drift in seasonal influenza to guide vaccine strain selection, demonstrating that DL and phylogeny-based models can be generalized to address the computational challenges posed by fast-evolving RNA viruses. Comparable computational strategies are applied to HIV to forecast drug-resistance mutations and viral fitness landscapes, while ML models grounded in sequence information are being pursued to trace genotype-phenotype relationships in HPV and dengue virus clades (Shi et al., 2025). These multi-viral examples show that AI-based mutation prediction models are not virus-specific and can be broadly applied across other viral families also with distinct evolutionary dynamics. Viral genomic datasets can be used to train AI models that analyze mutation hotspots, forecast evolutionary trajectories, and track the emergence of novel variants. Nonetheless, the rapid evolution of viruses can induce dataset shift and model drift, requiring periodic retraining and uncertainty-aware deployment strategies to maintain diagnostic reliability and high accuracy over long-term horizons (Choi et al., 2024).

### 3.1 Limitations of AI methodologies in virology

Although machine learning and deep learning models have been shown to exhibit a high level of performance in controlled benchmark datasets, several methodological limitations hinder their level of clinical reliability. Training data are frequently biased against certain groups of people, laboratory conditions, or variants of the virus with a decreased generalizability at real-world usage. Performance can also be worsened with time due to distribution shift due to viral evolution, sensor drift, and sampling protocol alterations. In other cases, most AI models do not have calibrated uncertainty estimates, which leads to larger overconfident predictions in the case of unseen variants or any biosensor signal noise. These limitations underscore the need for uncertainty-aware modeling, external validation across heterogeneous cohorts, and prospective clinical evaluation before deployment in diagnostic workflows (Mennella et al., 2024).

## 4 Nanotechnology-based viral detection approaches

Nanotechnology is changing the field of virology thanks to its ability to handle materials at the smallest levels, making it possible to detect viral pathogens in minutes. Typically, regular diagnostic methods need amplification, marking, or lots of sample preparation, but nanotechnology-based methods give affordable and portable options (Pradhan et al., 2021). Many researchers utilize nanosensors to detect viral components such as proteins, nucleic acids, or entire virions. These nanosensors, which often operate through electrochemical or optical mechanisms, typically incorporate materials like gold nanoparticles, carbon nanotubes, or graphene to enhance signal detection, enabling the rapid identification of viruses even in trace amounts (Sargazi et al., 2022).

Quantum dots (QDs), which are semiconductor nanocrystals known for their optical advantages, have opened new opportunities in nanotechnology diagnostics. If attached to antibodies or aptamers, QDs give off bright and steady fluorescence after attaching to various strains of viruses, which helps detect many viruses at the same time. This is most helpful with respiratory panels, because co-infections with influenza, RSV, and coronavirus are very common (Pareek et al., 2025). In a similar manner, Surface Plasmon Resonance (SPR) and Localized Surface Plasmon Resonance (LSPR) uses plasmonic colloids, such as gold and silver particles, to quickly watch over biomolecular interactions, ensuring that sensitive detection happens without involving labels (Takemura, 2021). Tools called lab-on-a-chip use microfluidics and nanomaterials to merge sample analysis, amplification, and detection into one simple device, helping virology enormously. These systems are capable of processing different methods in diagnostics such as lysis, extracting RNA, transcribing RNA, and reading the results with swift speed. When supported by either mobile readers or AI, LOC platforms can perform diagnostics in both rural and poor regions. AI is now being integrated with nanodevices to allow these devices to keep learning and adjust their settings, further reducing false positives and enhancing robustness (Zhu et al., 2020).

Aptamer-based nanosensors have provided better ways to find and detect specific viral proteins. This approach protects them from viruses that evolve the ability to evade hosts. Researchers have also made use of magnetic nanoparticles (MNPs) to help separate and extract viruses from large and complex biological samples prior to analysis. They are also used in testing and cool devices designed for checking and monitoring viruses in air and in the environment. Viral aerosols in closed places such as hospitals, airports, and transport systems can be picked up by filters with nanoscale sensors (Lou et al., 2022). A summary of nanotechnology platforms and their AI-integrated diagnostic functions is provided in (Table 2). With AI-enabled security systems, these filtration methods can quickly detect community cases of infectious disease. Viruses are detected quickly and with great sensitivity and easily transported, thanks to such systems, making them highly important in the fight against infectious diseases. With the progress of these technologies, it will be easier to address viral concerns before they develop into pandemics (Cozzolino et al., 2025).

**TABLE 2:** Nanotechnology-based viral detection platforms and their AI integration approach.

Nanotech platform	Target virus/Application	AI role	Advantages	Reference
Gold Nanoparticles (AuNPs)	SARS-CoV-2, Influenza virus detection	Enhances colorimetric signal interpretation	Offers quick, visible, and low-cost diagnostic tools	Pradhan et al. (2021), Cozzolino et al. (2025)
Carbon Nanotubes	Detection of HIV, Zika	Supports sensor signal filtering	High electrical conductivity improves detection sensitivity	Choi et al. (2024), Mokhtarzadeh et al. (2017)
Graphene Biosensors	COVID-19 antibody/antigen detection	Enables real-time analytics at the edge	Delivery ultra-sensitive results rapidly and portably	Sargazi et al. (2022), Tripathy et al. (2024)
Quantum Dots	Multiplex detection of multiple viral strains	Image classification, fluorescence analysis	Allows multi-pathogen analysis with high resolution	Mennella et al. (2024), Valenzuela-Amaro et al. (2023)
Magnetic Nanoparticles	Viral RNA/DNA extraction and purification	Drives sorting, data handling optimization	Enhances speed and purity of sample concentration	Pareek et al. (2025), Draz et al. (2020)

Nanotechnology-enabled viral diagnostics have also been developed for non-respiratory viruses, highlighting the platform-agnostic nature of nanosensing technologies. Electrochemical biosensors, graphene-based biosensors have been investigated to detect HIV antigen as well as nucleic acid in blood and saliva samples and nanoparticle-based as well as quantum dot-based tests have been developed to detect dengue virus or HPV biomarkers in blood. The same is also demonstrated by multiplexed nanosensor platforms (i.e. simultaneous detection of influenza, RSV, and SARS-like viruses), which can just perform a re-purposing of an existing nanotechnology platform without significant hardware alterations (Misra et al., 2022).

## 5 Integration of AI and nanotechnology for virus detection

The merging of AI with nanotechnology creates a major advance in virology by giving us unique tools for precise diagnostics and continual monitoring. In certain situations, AI can examine the fluorescence from QD molecules or the changes in impedance from nanoscale transistors and pick out the presence of viruses. They become especially important for spotting viruses that are hard to find and also for detecting them early in the infection process (Naik and Jagtap, 2024).

This combination will likely lead to the development of AI-based Point-of-Care (POC) devices that make use of nanomaterial detection platforms. They are effective in undertaking quick diagnoses in locations that cannot access main laboratories. Embedded AI enables adaptive calibration, signal denoising, and real-time interpretation of nanosensor outputs (Wasilewski et al., 2024). With this arrangement, communities can be monitored in real time, allowing faster reactions in the healthcare sector. AI plays a key role in facilitating nanosensors that are easier to interpret and more reliable by separating true viral signals from background noise and reducing false positives. CNNs can analyze nanoparticle assay images to detect subtle binding interactions not readily discernible by manual inspection (Goumas et al., 2025).

Integrated AI frameworks enable fusion of nanosensor outputs with clinical and environmental data streams to contextualize detection signals and support outbreak situational awareness.

**FIGURE 2:**
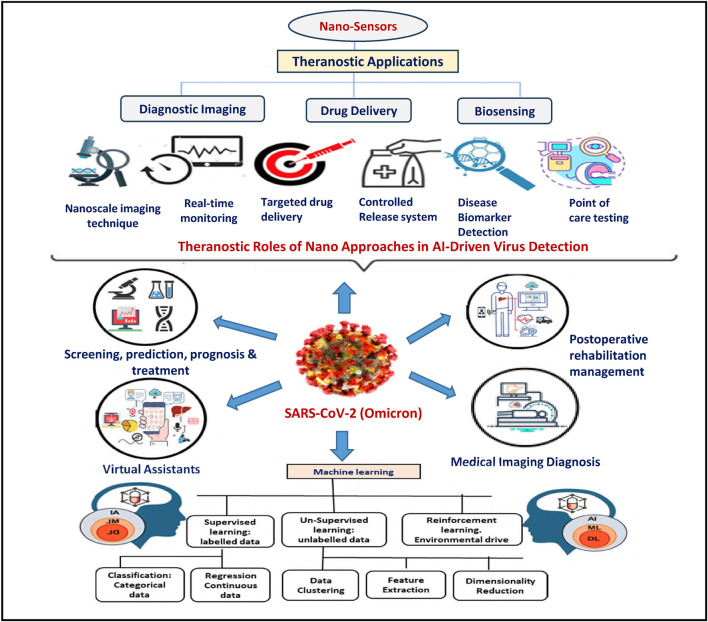
Theragnostic roles of nano-approaches in ai-driven virus detection. This figure illustrates the integration of nanotechnology and AI in virus detection, highlighting the theragnostic applications of nanosystems. Nanoparticles are utilized in biosensing, molecular diagnostics, targeted delivery, and real-time monitoring, while AI algorithms analyze complex biological data to enhance detection accuracy, predict disease progression, and guide treatment strategies.

For example, a nanosensor designed to detect respiratory viruses can be coupled with AI systems that monitor air quality and clinical symptoms, providing a holistic picture of an emerging outbreak. Wearable nanosensors can continuously monitor biomarkers and transmit real-time data to AI systems for dynamic risk stratification and early warning (Bhange and Telange, 2025). Applying AI together with nanotechnology is set to shape the methods we use to detect, monitor and respond to threats from viruses. As these two areas keep changing, their combination will encourage the creation of intelligent and autonomous tools that are essential for global health security (Draz et al., 2020). The multifaceted theranostic applications of nanotechnology integrated with AI in virus detection are illustrated in Figure 2.

Despite the wide range of AI-applied nanotechnology systems that have shown proof-of-concept performance in laboratory and pre-clinical studies, only a small number have been prospectively tested in clinical and regulatory settings. As a result, reported performance metrics are often highly contextual and may not reflect how durable these systems will be in heterogeneous, real-world environments. This Perspective therefore distinguishes between experimental-level prototypes, early translational devices, and clinically validated diagnostics, all of which should be benchmarked using evidence-based performance criteria before being considered for routine use.

### 5.1 Operational workflow of AI–nanotechnology integration for viral diagnostics

Beyond the conceptual integration, the AI–nanotechnology pipeline can be implemented as a practical, multistage workflow that includes sensing, computation, validation, and deployment. To start with, nanotechnology-based systems are used to capture biological samples (e.g., saliva, blood, breath condensate, and wastewater). These include electrochemical nanosensors, plasmonic SPR/LSPR chips, graphene transistors, and quantum dot–based fluorescence assays. They detect viral binding events and convert them into digital signals such as impedance changes, fluorescence pattern images, or spectral profiles (Akpan, 2025).

Second, the raw nanosensor data are preprocessed; that is, noise is filtered out, baselines are corrected, signals are normalized, and time alignment is performed. Depending on the data modality, feature extraction can rely on CNNs for image-based nanoparticle or fluorescence readouts; LSTM networks or Transformer models for time-series streams from continuous biosensors; and classical ML models such as RFs and SVMs for structured electrochemical data (Hoque et al., 2020).

Third, model training and validation are carried out using labeled datasets obtained from verified RT-PCR or sequencing results, allowing AI predictions to be supervised and clinically grounded. Continual learning strategies and regular model retraining with newly acquired data help reduce model drift caused by viral mutations or sensor degradation over time. Through domain adaptation, models developed under controlled laboratory conditions can be tuned and transferred to real-world field or point-of-care settings (Tungal et al., 2025).

Fourth, edge deployment enables near real-time inference on portable point-of-care devices or smartphone-based readers, reducing latency, and minimizing reliance on centralized cloud computing. AI-assisted signal interpretation improves sensitivity and specificity, strengthens overall performance, and helps distinguish true viral signatures from environmental noise and non-specific binding (Wang et al., 2025).

Finally, a clinical feedback loop is introduced, where results from confirmatory diagnostics and clinical follow-up are fed back into the training pipeline. This allows the system to continuously refine and adapt its performance over time. Together, this hands-free AI–nano design transforms nanosensors from passive detectors into learning diagnostic tools that improve in accuracy as more data are collected and new viral variants emerge (Bhaiyya et al., 2024).

### 5.2 Viral detection: representative case studies of AI–nanotechnology integration

To demonstrate the practical feasibility of AI convergence with nanotechnology in real developmental settings, a few representative cases across viral systems are discussed below:

#### 5.2.1 Case study 1: smartphone-integrated nanoparticle sensors for respiratory virus detection

Optical biosensors that use nanoparticles have been integrated with deep neural networks to enable the detection of respiratory viruses with the assistance of a smartphone. In these systems, gold or fluorescent nanoparticles functionalized with viral probes produce colorimetric or fluorescence patterns following viral binding. CNNs, trained on image samples captured by smartphone cameras, are sensitive to subtle intensity and spatial variations that are not visible to the human eye, which enhances sensitivity and minimizes false positives (Draz et al., 2020). These strategies have shown that it is possible to build low-cost, field-deployable detection pipelines that can be used for respiratory virus surveillance in community and resource-constrained environments.

#### 5.2.2 Case study 2: ML–based electrochemical nanosensors for blood-borne virus detection

Graphene or carbon nanotube (CNT) based electrochemical nanosensors generate high-dimensional electrochemical impedance and current–voltage signatures when viral antigens or nucleic acids bind to the sensor surface. These multivariate electrochemical features have been analyzed using RF and SVM models to discriminate between infected and non-infected samples, showing improved resistance to sensor noise and environmental variability. These ML-based nanosensors offer rapid point-of-care screening of blood-borne viruses and illustrate how classical ML models remain useful for low-dimensional, structured biosensor outputs (Özmen et al., 2021).

#### 5.2.3 Case study 3: DL and time-series bio-sensing for arbovirus surveillance

Temporal biosignatures of viral exposure patterns are produced by continuous measurements from environmental or wearable nanosensors. These biosensor time-series streams have been analyzed using LSTM networks and Transformer-based models, allowing the identification of temporal relationships linked to early infection dynamics and outbreak onset. These approaches enable probabilistic assessment of vector-borne virus occurrence by integrating biosensor data with environmental conditions, including temperature, humidity, and patterns of population movement, among others (Zhang et al., 2025).

#### 5.2.4 Case study 4: AI-improved multiplex detection of Co-Circulating viruses

QD–based nanosensors can detect more than one viral target simultaneously within the same assay by multiplexing fluorescence signals. CNN-based multi-label classification schemes have been applied to spectral and imaging data from multiplexed nanosensor systems to classify co-infections and overlapping signal patterns. This technique is particularly relevant for screening viruses with overlapping clinical phenotypes that are also co-circulating, such as in respiratory panels, enabling unified screening within a single point-of-care test (Mousavi et al., 2022) (Table 3).

**TABLE 3:** Challenges and future directions of AI–nanotechnology integration in viral prediction.

Challenge	Underlying issue	Potential AI/Nano solution	Expected impact
Data Privacy & Security	Sensitive health/genomic data at risk	Block-chain-based storage, federated learning	Secure, reorganized data management
Algorithmic Bias	Limited diversity in training datasets	Use of balanced, multi-ethnic global datasets	Fair predictions across populations
Technical Integration	Lack of standardized platforms for AI-nano conjunction	Development of interoperable frameworks	All-in-one integration and scalability
Biosafety Concerns	Unclear long-term toxicity of nanomaterials	Biodegradable/eco-friendly nanomaterials	Safer deployment in healthcare
Accessibility	High infrastructure demands in low-resource regions	Portable AI-driven nanosensors, mobile-based POC kits	Democratized viral diagnostics
Regulatory Barriers	Absence of universal standards for AI–nano devices	Global harmonization of regulatory guidelines	Faster clinical translation
Future Prospects	Need for advanced computing support	Quantum ML, neuromorphic computing	Energy-efficient and ultra-fast analysis

Altogether, these examples illustrate that AI models are not generic add-ons but are selected based on the data modality (image, electrochemical signal, time-series stream) and the clinical implementation context. These case studies also help define the translational gradient between laboratory prototypes and near-clinical point-of-care implementations, not only showing that the tools are scalable but also identifying the remaining roadblocks along the way. To further underscore the virus-agnostic, real-world applicability of AI–nanotechnology convergence across respiratory, blood-borne, sexually transmitted, and agent-to-host pathogens, Table 4 summarizes representative applications involving influenza, HIV, HPV, dengue, and RSV.

**TABLE 4:** Illustrative virus-specific use cases of AI–nanotechnology integration.

Virus system	Sample type	Nanotechnology platform	AI model application	Clinical/Surveillance relevance
Influenza	Nasal swab	Plasmonic or QD nanosensors	CNN-based signal/image classification	Vaccine strain forecasting, outbreak surveillance
HIV	Blood/saliva	Graphene/electrochemical biosensors	RF/SVM for antigen/nucleic acid classification	Point-of-care screening, treatment monitoring
Dengue	Blood	Nanoparticle fluorescence assays	CNN/LSTM for early infection signal detection	Vector-borne outbreak monitoring
HPV	Cervical samples	Quantum dot multiplex assays	Multi-label CNN classification	Cancer risk screening
RSV	Nasal swab	Lab-on-chip nanosensors	CNN-based pattern recognition	Pediatric respiratory diagnostics

### 5.3 Flaws of AI models in the case of rapid viral mutation and model drift

Even though AI models have the capability to learn trends in viral genomes, antigenic profiles, and biosensor signal patterns, the sheer speed and unpredictability with which many viruses mutate remains a major challenge for the long-term reliability of these models. With viral evolution, the training data and real-world deployment conditions begin to differ, leading to a distribution shift. This results in model drift and a gradual deterioration of diagnostic performance over time. For instance, changes in viral epitopes can alter nanosensor binding affinities or spectral signals, which in turn reduces the precision of AI models trained on earlier viral variants. This type of dataset obsolescence is a key limitation when deploying static AI models during outbreaks, especially in the case of rapidly evolving RNA viruses (Guan et al., 2025).

To reduce these risks, regular learning and routine retraining on newly generated clinical and sequencing data are needed to preserve diagnostic strength. The generalization of models trained in laboratory settings can also be improved through domain adaptation strategies when transferring them to field or point-of-care environments. Moreover, AI-assisted diagnostic pipelines should be complemented with uncertainty-aware models and confidence calibration so that low-confidence predictions can be flagged when new variants or previously unseen signal patterns appear. These precautionary measures are essential to avoid unnecessary dependence on outdated models and to ensure safer clinical implementation of AI technologies in nanotechnology under evolving viral conditions (Javed et al., 2025).

### 5.4 Reconsidering major assumptions in AI–nano viral diagnostics

One common belief in the field is that overall gains in the accuracy of AI models or improvements in nanosensor sensitivity have a directly proportional relationship with clinical readiness. However, performance gains achieved in controlled laboratory settings often do not translate well to less homogeneous real-world environments because of dataset shift, sensor drift, population heterogeneity, and the continuous emergence of changing viral variants (Zhao et al., 2025). This creates a clear discontinuity between proof-of-concept demonstrations and truly deployable diagnostic frameworks.

Another assumption that needs to be reconsidered is that translational success is driven mainly by algorithmic sophistication. In practice, regulatory validation, standardized manufacturing of nanomaterials, long-term stability, calibration of nanosensors, and governance and monitoring of AI models after deployment are equally limiting factors. These practical constraints often determine whether technology can move from the lab to real clinical use. To overcome these bottlenecks, the integration of AI and nanotechnology should be viewed as a continuously validated clinical system rather than a one-time technological object (Kelly et al., 2019). In this perspective, there is a need to move beyond performance-based benchmarking alone toward lifecycle-aware evaluation frameworks that include clinical validation, alignment with regulatory requirements, and long-term monitoring and maintenance of deployed models.

### 5.5 Translational barriers in AI–nano viral diagnostics

Regardless of positive laboratory findings, nanotechnology-based viral diagnostics face several non-trivial limitations when being translated into clinical and public health services. The biosafety of long-term effects is still not fully defined for many nanomaterials, including concerns around nanoparticle stability, bioaccumulation, immunogenicity, and off-target effects, especially in the case of wearable or implantable sensing devices. Scalability and reproducibility of manufacturing also remain major challenges. Variations in nanoparticle synthesis, surface functionalization, and device fabrication can lead to batch-to-batch performance differences, which complicate quality control and regulatory approval (Thwala et al., 2023).

Standardization across nanosensor platforms is still not a practical reality, and harmonized protocols for calibration, benchmarking, and inter-laboratory performance comparison are largely lacking. In addition, the co-validation of AI model software and hardware components (nanomaterial-based sensors) further complicates regulatory approval for AI- or nanotechnology-driven diagnostics. The frequent updates required to adapt AI models to viral evolution and sensor drift also challenge conservative regulatory frameworks that were originally designed for relatively inert and static medical devices (Chaudhary and Bhadola, 2025). Altogether, these considerations show that technological innovation alone is not sufficient for successful translation. Regulatory co-design needs to begin early in development, and standardized manufacturing pipelines must be supported by continuous post-deployment performance monitoring and governance.

## 6 AI for outbreak prediction and smart epidemiological modeling

AI plays an essential role in predicting and controlling viral outbreaks by providing quick and accurate responses to such situations. With data from clinical records, internet trends, global movements, and monitoring stations, AI is able to find gentle changes that might reveal the presence of infectious diseases. Decision trees, RFs, and SVMs are some of the ML algorithms that have helped researchers examine past data from outbreaks and predict how the virus would move forward by considering factors such as host behavior, density, and weather changes in a population (Mokhtarzadeh et al., 2017; Tripathy et al., 2024). Results have shown that RNNs and LSTMs are very effective at forecasting infections and how they spread geographically. They are able to handle sequential information about infections and consider how it may take time for effects to be seen, and that diseases often affect each other in unusual ways. For example, AI dashboards based on ML were important in providing accurate projections of cases, the demand for beds in intensive care units, and death rates during the COVID-19 pandemic (Kamalov et al., 2022).

Reinforcement learning also adds value by simulating how different efforts to contain outbreaks might work in different situations. RL agents practice finding the best strategies for measures such as lockdown, testing regularly, or choosing vaccination schedules by improving the results experienced in simulations. Such simulations let decision-makers compare different situations in terms of health and the economy (Bushaj et al., 2022). In addition, combining AI models with spatial maps and current traffic data helps create detailed maps of possible dangers. The use of these graphs allows resources to be concentrated on key areas, groups at risk, and those that need immediate assistance. Mixing agent-based simulations and ML in the forecasting of outbreaks makes these tools more accurate and flexible (Hochmair et al., 2025). Using AI with wearable health gadgets and sensors in the environment can spot warning signs of infection earlier than regular medical tests. Analyzing these data streams through AI methods gives advance notice about possible outbreaks and improves the speed of action. It is also worth noting that AI makes it easier to track misinformation and observe people’s actions during pandemics (Shaj et al., 2023). NLP tools review what people in the public determine and how rumors spread, offering information about influences on the development of an outbreak. To sum up, AI-driven outbreak prediction and modeling are shifting public health readiness. When models are more understandable, open, and bring together various data sources, using them in policy and emergency response will play a major role in dealing with future viruses (Al-Garadi et al., 2022).

### 6.1 Clinical translation, pathways to validation, and implementation readiness of AI–nano diagnostics

Although there has been fast advancement in AI-based nanosensor platforms, most reported systems are still at the proof-of-concept stage or limited to early laboratory testing. Several interdependent obstacles must be addressed before these technologies can move into routine clinical or public health use. These include technical validation, regulatory approval, manufacturability, and reliable ways to monitor post-deployment performance. Nanosensor validation (sensitivity, specificity, limit of detection, and robustness to environmental variability) should be assessed against gold standards such as RT-PCR or sequencing, ideally across multi-center clinical cohorts. ML models trained on laboratory-curated datasets often face dataset shift when applied to real-life, heterogeneous settings (Subbaswamy and Saria, 2025). As a result, performance can drop across different demographic groups, sample matrices, and viral strains, especially when external populations or sample types are not adequately validated. Future clinical studies are therefore needed to demonstrate non-inferiority or superiority compared with current diagnostic standards in real-world clinical workflows.

Regulatory approval presents another major challenge because both nanomaterials and adaptive AI algorithms are novel, and their combination is even newer. Unlike traditional diagnostic systems, AI-powered platforms can update over time, which raise concerns around regulatory re-certification, version control, auditability, and algorithm transparency (Rosenthal et al., 2025). Regulatory acceptance will require standardized reporting structures for AI–nano diagnostics, including dataset provenance, performance reporting guidelines, model update protocols, and post-market surveillance frameworks.

Another key translational bottleneck lies in biosafety and scalable manufacturing. While nanosensors often show high sensitivity in laboratory settings, reproducible large-scale fabrication with consistent surface functionalization remains non-trivial. Batch-to-batch variability can lead to signal drift over time, which in turn affects AI model performance. Moreover, although short-term biocompatibility of nanomaterials in disposable diagnostics appears promising, the long-term biocompatibility and environmental impact of these materials remain insufficiently evaluated through systematic studies (Subasri et al., 2025).

Finally, sustainable real-world implementation will require infrastructure to support continuous learning and post-deployment feedback. AI-based models deployed in point-of-care devices need frequent recalibration to adapt to viral evolution, sensor degradation, and changing epidemiological dynamics. Adaptive adjustment of diagnostic performance, while respecting data governance and privacy constraints, can be supported through clinical feedback loops, federated learning approaches, and privacy-preserving data-sharing frameworks (Kim et al., 2024).

Taken together, these translational considerations suggest that AI–nano diagnostics should not yet be viewed as fully mature diagnostic solutions, but rather as emerging clinical pipelines. To place the current state of the art in perspective, Table 5 highlights key development milestones, validation stages, and critical roadblocks across laboratory prototypes, pilot clinical studies, and near-clinical platforms.

**TABLE 5:** Translational readiness levels of representative AI–nano diagnostic platforms.

Development stage	Typical characteristics	Validation status	Key bottlenecks
Proof-of-Concept (Lab)	High sensitivity under controlled conditions; small curated datasets	Bench validation only	Dataset shift, reproducibility, lack of clinical cohorts
Pilot Clinical Evaluation	Tested on limited patient samples; early comparison with RT-PCR	Single-center or small cohort studies	Regulatory pathway unclear, device standardization
Near-Clinical Prototype	Integrated hardware–software pipeline; point-of-care format	Multi-site feasibility testing	Manufacturing scale-up, regulatory approval, model governance
Deployment-Ready (Target State)	Validated across diverse populations; post-market monitoring	Large-scale clinical trials	Cost, supply chain, long-term AI maintenance

## 7 Challenges and ethical considerations

Connecting AI and nanotechnology to fight against viruses has great advantages, yet serious challenges and ethical matters are involved. Data privacy is one of the greatest concerns in the field (Murdoch, 2021). AI systems depend on collecting huge amounts of personal and biological details frequently obtained by wearable gear, genome repositories, and mobile programs, which makes some individuals worry about having their data copied without permission or used for the wrong reasons (Yadav et al., 2023). To care for people’s privacy and have their trust, it is crucial to obey regulations such as GDPR and HIPAA. Algorithms can inherit unintentional bias, which adds another major challenge. If the data used to train AI models is not diverse and does not include everyone fairly, its forecast and analysis can result in unfairness in the delivery of medical care to various populations (Hanna et al., 2025). To handle this, it is necessary to create datasets that represent all individuals, use fair and transparent ML tools, and apply clear verification processes (Ueda et al., 2024). A consolidated overview of these challenges, along with potential technological and methodological solutions, is presented in (Table 3).

Experts in nanotechnology are always concerned about biosafety and how it impacts the environment. We are still in the process of finding out about the lasting impacts of nanomaterials on humans and the environment. Rules need to change as nano-enabled devices appear more and more in both medical and consumer areas. It is often difficult to achieve smooth integration because of various technical issues (Ray et al., 2009). Data from AI needs to match the format used by nano-biosensors, processes should be done on the fly, and systems must remain secure in changing environments. The absence of standard platforms or compatible frameworks usually leads to systems that are not easy to grow (Arya et al., 2023). Moreover, it is difficult for low-resource countries to use advanced AI and nano technology because of the infrastructure needed. It is very important to connect everyone worldwide to these digital tools so that health equality is global and these technologies can be fully used (Zuhair et al., 2024). In summary, some people worry about the overall impact of AI systems on decision-making in healthcare. Issues concerning who is responsible, how decisions are explained and the proper balance between human control and machine actions need to be studied by working together with ethicists, technologists, doctors, and policymakers. If stakeholders are ready for these issues and deal with them upfront, AI and nanotechnology can develop virology in ways that put safety, fairness, and benefits above everything (Mennella et al., 2024). Beyond technological innovation, the central challenge for AI–nanotechnology viral diagnostics lies in bridging the translational gap between laboratory prototypes and regulated clinical tools. This Perspective advances the view that future progress will be constrained less by algorithmic novelty and more by the development of lifecycle-aware diagnostic systems that incorporate continual learning, variant-aware recalibration, regulatory co-design, and post-deployment governance. Recognizing and addressing these overlooked bottlenecks is essential to prevent repeated cycles of promising proof-of-concept studies that fail to achieve sustained clinical impact.

## 8 Future prospects and research directions

**FIGURE 3:**
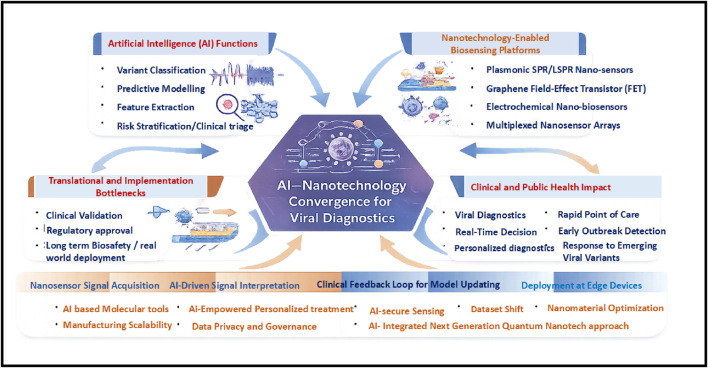
Conceptual framework of AI–nanotechnology convergence for viral diagnostics and surveillance. Conceptual overview illustrating the synergistic roles of AI and nanotechnology in viral diagnostics, linking nanosensor-derived biosignatures to AI-driven functions (signal denoising, feature learning, variant classification, uncertainty estimation, and risk stratification). The framework integrates translational and implementation constraints, including clinical validation, regulatory approval, manufacturing scalability, dataset shift, and data governance, and maps these technical capabilities to clinical and public health impact (point-of-care testing, surveillance, and adaptive outbreak response). This figure emphasizes AI–nanotechnology convergence as an emerging translational pipeline rather than a fully mature clinical solution.

The integration of AI and nanotechnology has the potential to reshape how infectious diseases are detected, monitored, and managed globally. Improving the interpretability and transparency of AI models will be essential for regulatory approval and clinical adoption, particularly in safety-critical diagnostic workflows (Shirzad et al., 2025). Advances in neuromorphic computing and quantum ML may further enhance the speed and energy efficiency of AI–nano platforms (Al Abdul Wahid et al., 2024). The new generation of nanosensors is being designed to be more biocompatible, multifunctional, and self-sustaining, eliminating the need for an external power source. These advanced nanosensors can autonomously collect samples, convert ambient energy, and be integrated into wearable or implantable devices (Ramesh et al., 2023). Emerging technologies such as DNA origami, nanozymes, and plasmonic materials are currently under investigation for their potential to detect and respond to biological signals or pathogens in real time (Valenzuela-Amaro et al., 2023). The future scope of AI–nanotechnology integration across nanosensor platforms, nanomaterial design, quantum technologies, and personalized treatment planning is schematically summarized in Figure 3.

Long-term improvements will be associated with intensive interdisciplinary partnership between data and virologists, engineers, clinicians, and public health specialists in building clinically relevant and operationally deployable systems (Lv et al., 2024). Multi-omics information (genomics, proteomics, transcriptomic) combined with AI-based bio-sensing can potentially allow closer and, possibly, more personalized viral risk assessment and outbreak prediction. In case the data governance system and privacy frameworks are strong, decentralized digital health models may grow an opportunity to early-stage detection within the community and resource-restricted contexts. Distributed surveillance systems can be improved with block-chain-based data infrastructures to provide greater levels of transparency and traceability (Sah et al., 2025).

Implementation of AI-nanotechnology diagnostics in the clinical and population contexts of practice is going to necessitate joint development of biosafety tests, scalable manufacturing, standardization of the platform, and regulation such that adapting algorithms can be allowed. Until these systemic limitations are incorporated, it is doubtful that many good laboratory prototypes will go beyond demonstrations of proof-of-concept. Based on these points, the majority of the suggested AI-nano workflows are conditional upon strict clinical validation, regulatory authorization, manufacturing preparedness, and long-term post-implementation performance metrics (Vora et al., 2023).

## 9 Conclusion

AI being combined with nanotechnology marks a big change in how we can spot and prepare for viruses. We have seen in this review that AI can analyze trends and make predictions, while nanotechnology can track down pathogens with precision and great sensitivity. With the help of these technologies, there is a strong framework in place to improve both virology and strengthen public health (Nandipati et al., 2024). Early achievements look very hopeful, as there are already platforms and prototypes that have tested the use of AI–nano systems in daily situations. Still, using these systems around the world will require dealing with technical, social, legal, and infrastructure issues. AI, nanomaterials, and biotechnology should be used in a fair way, with all algorithms made clear and following all safety measures (Bachmann et al., 2022).

Although COVID-19 intensified the speed of AI–nanotechnology convergence, the modes described across here are virus-agnostic structures and can be generalized to a large number of various viral pathogen types, namely respiratory viruses, blood-borne viruses, sexual infections, and multifocal viruses. The ability to scale AI- nano of a wide range of viral infrastructures highlights the opportunities of using such systems as a generalized infrastructure in the future to prepare against outbreaks and as a routine tool to monitor infectious diseases. In the future, we look forward to AI identifying upcoming viral changes and outbreaks as well as sensors built on nanotechnology giving instant information from different types of samples. If guided by global effort and inclusive laws, these innovations may help us manage viruses effectively in the future. All in all, linking AI with nanotechnology is not only a technical breakthrough but also necessary for improving virology in an ever connected and threatened world. Further focus on research, teamwork among different specialists, and careful use of these advances will play a big role in ensuring global health (Ramalingam et al., 2023).

## Data Availability

The original contributions presented in the study are included in the article/supplementary material, further inquiries can be directed to the corresponding author.
